# Exploring the Frontiers of Mathematical Neuroscience: A Comprehensive Bibliometric Analysis

**DOI:** 10.7759/cureus.71213

**Published:** 2024-10-10

**Authors:** Jais Kurian, Dary John, Pratheesh Mathew, Liny Mariam Mathew, Jobin Jose

**Affiliations:** 1 Department of Mathematics, St. Stephen's College, Uzhavoor, Uzhavoor, IND; 2 Department of Mathematics, Newman College, Thodupuzha, IND; 3 Department of Mathematics, Nirmala College (Autonomous), Muvattupuzha, IND; 4 Department of Mathematics, Devaswom Board College, Thalayolaparambu, Thalayolaparambu, IND; 5 Department of Library Science, Marian College Kuttikkanam (Autonomous), Kuttikkanam, IND

**Keywords:** bibliometric analysis, biblioshiny, mathematical neuroscience, mathematics, neuroscience, vosviewer

## Abstract

Mathematical neuroscience is the branch of interdisciplinarity between mathematical modeling and neuroscience through computational techniques to study the structure, function, and dynamics of the brain. The objective of this paper is to undertake a comprehensive review of research trends in mathematical neuroscience and important developments in the period from 1973 to 2024. From this source of bibliographic data, Scopus alone returns 727 retrieved documents, consisting of journals, book chapters, and conference papers. The analysis showed an annual growth rate of 6.51% in this field and significant contributions from authors of 1957 sources. Specific tools were used in this review for in-depth analysis of publication patterns, co-authorship networks, keyword co-occurrences, and thematic evolution within this discipline. The most influential authors, dominant publication sources, and most active countries in the field were identified. The survey also underlines various other emerging trends, of which the highly increasing approach to the integration of machine learning and artificial intelligence (AI) with mathematical neuroscience is certainly the most interesting. Results highlight the dynamic and collaborative features of research in this area and provide insight into the intellectual landscape for research in this field, along with its future directions.

## Introduction and background

Mathematical neuroscience is a fast-growing field at the junction of mathematical, statistical, and computational methods aimed at uncovering complicated neural processes. In the past years, there has been prominent development and refinement of mathematical models describing the electrical activity of neurons at the individual level and across large neural networks. These models have removed the quite damning gap between computational neuroscience and statistical paradigms by providing a bridge between mechanistic theory and statistical paradigms, hence allowing integration that gives deeper insight into neural dynamics and their implications for behavior, as Kass et al. put it in their comprehensive review of this burgeoning field [1].

Another important direction in mathematical neuroscience is connected with applications of advanced mathematical methods for processing neurophysiological data, especially in the context of brain-computer interfaces (BCIs). Wavelet analysis and other new techniques introduced by Hramov et al. have shown that it is possible to decode information from non-stationary neurophysiological processes with time-varying features. These methods turned out to be very important for the analysis of neuronal network dynamics at all levels, from the micro-level of individual cells to the macro-level of large-scale brain activities [2]. Moreover, the design and development of mathematical models have made very prominent progress, thereby giving important tools in the understanding of brain functions. Another study explores the mathematical modeling of neurite growth and morphogenesis, emphasizing the role of biophysical effects such as elasticity, viscosity, and chemical signaling. This review critically examines various models for neurite development, focusing on those that can be analytically treated to uncover the underlying mechanisms driving neuronal network formation [3].

In cognitive neuroscience, mathematical models have played an equally important role in understanding cognitive development vis-à-vis the domain of mathematical abilities. Evolutionary developmental psychology provided a framework for examining how genetic, neural, and environmental factors interact in shaping these abilities. Brown's study epitomizes the need to combine neuroscience with educational practice to understand how the brain handles difficult mathematical concepts [4]. An area that has been growing within educational neuroscience, something that seeks to apply brain-related findings to improve learning processes, concerns ways to enhance mathematics education. According to Antonopoulou et al., knowing the neural basis of mathematical processing can result in more effective teaching strategies and educational outcomes [5].

Some of the emerging trends in mathematical neuroscience relate to the growing interface between artificial intelligence (AI) and neuroscience. Recent work has initiated the exploration of how AI can model brain processes, particularly in terms of spatial navigation and reinforcement learning. Bermudez-Contreras et al. proposed that this interdisciplinary approach could foster advances in both areas by using insights from one field to gain a better understanding of neural computation and behavior [6]. Another area that is more promising in this field may be the application of predictive coding models in clinical neuroscience. In this case, such views on the brain are already used as a "prediction machine" for studying psychiatric disorders with new potentials for diagnostics and treatment. Work by Smith et al. itself especially accentuates the possibility of applying these models in clinical practice [7]. It is a time of change in the field of mathematical neuroscience, with sophisticated models and methods allowing new and perhaps deeper insights into brain functioning. However, it is the integration of these mathematical approaches with computational and cognitive neuroscience, along with applications for improving education and AI, that lays out a setting for major innovations in understanding the brain and brings improved mental health outcomes.

One such robust quantitative measure is bibliometric analysis, which can be used to gauge and analyze trends in academic literature and research across a wide array of fields [8-12]. That is a method that will allow patterns to emerge from a systematic review of publications, citations, and other scholarly outputs and, at the same time, pinpoint key works and map out the intellectual framework that shapes a discipline [13-16]. Biblioshiny is a web-based, user-friendly interface to the R-based bibliometric package that offers researchers an advanced environment to execute detailed analysis and visualization, not requiring extensive programming skills [17-21]. On the other hand, VOSviewer is an extensively applied software in the construction and analysis of bibliometric networks about co-authorship, co-citation, and keyword co-occurrence; it has turned out to be a great tool for providing valuable insight through visualization into complex research domains [22-26].

The primary objective of this study is to provide a comprehensive review of the research landscape for mathematical neuroscience from 1973 to 2024. The study explores key trends, influential authors, and significant publications that shaped the development of this domain at the intersection of disciplines. Specifically, this research in the field aims to unravel the newly emerging intellectual structure and the network of collaboration characterizing mathematical neuroscience. Moreover, the research will bring out emerging topics along with pointers toward future directions, something that will provide significant insight not only for researchers but also for practitioners and policymakers in the field of mathematics and neuroscience.

## Review

Materials and methods

**Figure 1:**
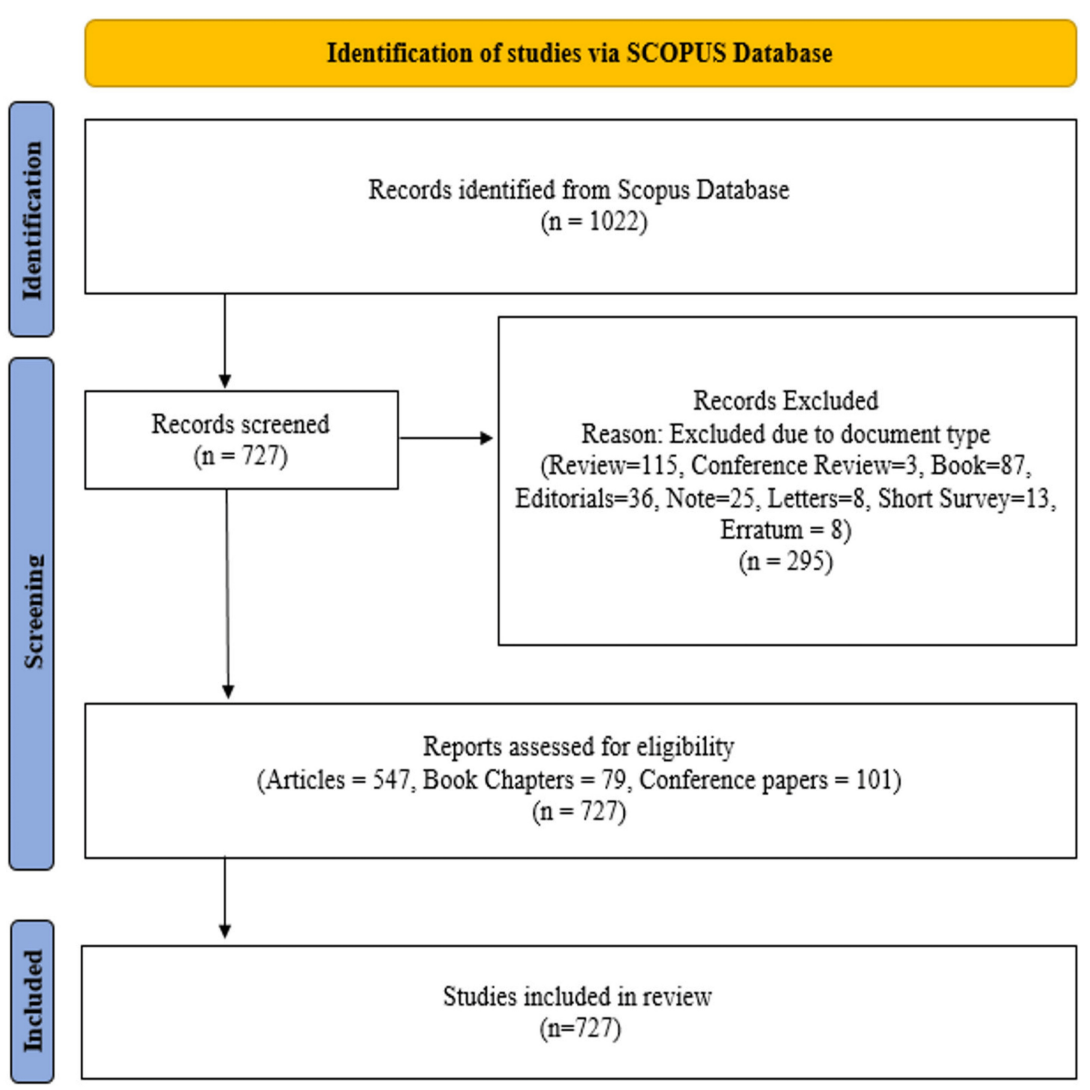
PRISMA Flow Diagram Illustrating the Selection Process for Studies Included in the Review PRISMA: Preferred Reporting Items for Systematic Reviews and Meta-Analyses

In this study, Scopus was chosen as the main source of bibliographic data due to its extensive collection of high-quality journals, offering broader coverage compared to other databases. Publications were retrieved using the keywords "Mathematical Neuroscience," OR "mathematics," AND "neuroscience," with no language restrictions, focusing specifically on journal articles, book chapters, and conference papers. The search yielded a total of 727 documents from 477 different sources, spanning the period from 1973 to 2024. Figure 1 illustrates the PRISMA methodology used for paper selection in the bibliometric analysis, which followed a three-step process. The first step involved identifying and extracting relevant data from the databases. The second step was to exclude reviews, editorials, letters, notes, and short surveys, refining the selection to articles, book chapters, and conference papers. The data was then saved as a "CSV" file, and the subsequent bibliometric analysis was performed using VOSviewer and Biblioshiny software.

Results

Table 1 presents a comprehensive overview of the key findings from the bibliometric analysis of mathematical neuroscience, covering the timespan from 1973 to 2024. The analysis reveals a steady annual growth rate of 6.51% in the field, with a total of 727 documents sourced from 477 different journals, books, and other publications. These documents have an average age of 9.74 years and are cited, on average, 23.18 times per document, indicating a strong impact within the academic community. The study identifies 4,177 Keywords Plus (ID) and 2,269 Author's Keywords (DE), reflecting the diverse topics and trends explored within this domain. With contributions from 1,957 authors, including 216 single-authored documents, the analysis highlights the collaborative nature of research in this field, as evidenced by the average of 2.97 co-authors per document and an international co-authorship rate of 20.36%. The document types predominantly include 547 articles, followed by 101 conference papers, and 79 book chapters, illustrating the various mediums through which research is disseminated.

**Table 1:** Key Information and Characteristics of the Investigated Studies

Description	Results
Main information about data	
Timespan	1973-2024
Sources (journals, books, etc.)	477
Documents	727
Annual growth rate %	6.51
Document average age	9.74
Average citations per document	23.18
References	33,947
Document contents	
Keywords Plus (ID)	4,177
Author's Keywords (DE)	2,269
Authors	
Authors	1,957
Authors of single-authored documents	216
Authors collaboration	
Single-authored documents	234
Co-authors per document	2.97
International co-authorships %	20.36
Document types	
Article	547
Book chapter	79
Conference paper	101

Annual Scientific Production

**Figure 2:**
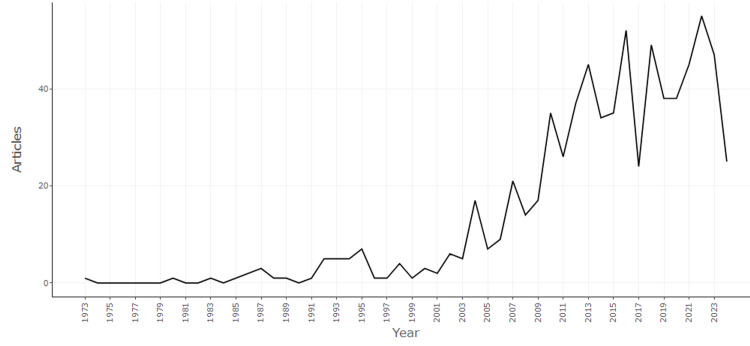
Annual Scientific Production: Yearly Distribution of Published Research Articles

Figure 2 depicts the annual scientific production in the field of mathematical neuroscience from 1973 to 2024. The data illustrates a significant increase in research activity over the years, particularly from the early 2000s onward. Initially, the number of articles published annually remained relatively low and stable, with minimal fluctuation until around 1999. However, from 2000 onward, there is a noticeable upward trend, indicating growing interest and advancements in the field. The number of articles published annually peaked several times, most notably around 2014, 2016, and 2020, when the production exceeded 40 articles per year. This peak suggests periods of intensified research activity, possibly driven by technological advancements or increased recognition of the importance of mathematical neuroscience. However, the most recent data shows a slight decline in the number of articles published in 2023, which may indicate a temporary dip in research output or a shift in focus within the field. Overall, the graph highlights the rapid growth and dynamic nature of research in mathematical neuroscience over the past few decades.

Most Relevant Authors

Table 2 highlights the most relevant authors in the field of mathematical neuroscience, showcasing their contributions through the number of articles published. Both Ansari D and Coombes S emerge as the leading contributors, each with eight articles, indicating their prominent roles and sustained research efforts in this area. Brown RD and De Smedt B follow with six articles each, demonstrating their significant impact on the field. Zhang L has contributed five articles, while Lee S-Y, Li X, Verschaffel L, and Wang X each have published four articles, reflecting their active participation and influence in the research community. Angelaki DE rounds out the list with three articles, adding to the collective body of knowledge in this domain. These authors represent the key thought leaders and researchers driving forward the exploration of mathematical neuroscience.

**Table 2:** Most Relevant Authors in Mathematical Neuroscience and Their Number of Published Articles

Authors	Number of articles
Ansari D	8
Coombes S	8
Brown RD	6
De Smedt B	6
Zhang L	5
Lee S-Y	4
Li X	4
Verschaffel L	4
Wang X	4
Angelaki DE	3

Authors' Production Over Time

**Figure 3:**
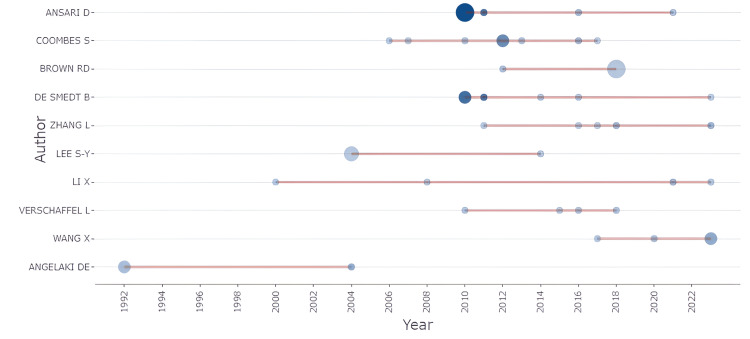
Temporal Trends in Authors' Research Output in Mathematical Neuroscience Circle size: It represents the number of articles published by each author in a given year. Larger circles indicate a higher number of publications. Circle color intensity: It reflects the total citations per year (TC per year), with darker shades indicating a higher number of citations.

Figure 3 illustrates the production of key authors in the field of mathematical neuroscience over time, highlighting the number of articles published and their total citations per year (TC per year). The horizontal axis represents the timeline, while the vertical axis lists the authors. The size of the circles corresponds to the number of articles published by each author in a given year, with larger circles indicating a higher number of publications. The color intensity of the circles reflects the total citations per year, with darker shades indicating more citations.

Ansari D and Coombes S are among the most prolific authors, with consistent publication records over several years, particularly from 2010 onward. Their work has also garnered significant citations, as evidenced by the darker circles. Brown RD and De Smedt B show similar patterns, although with slightly fewer publications. Authors such as Zhang L, Lee S-Y, and Li X have fewer but still notable contributions, with publications spread out over time and a focus on specific years. Verschaffel L and Wang X also have consistent but less frequent publication records. Angelaki DE is notable for having early publications (starting in the early 1990s) but with fewer recent contributions compared to others on the list. Overall, the figure provides a clear visual representation of each author's research activity over time, highlighting their impact through the number of publications and the citations those publications have received.

Most Relevant Sources

Table 3 presents the most relevant sources contributing to the field of mathematical neuroscience. The "Lecture Notes in Computer Science," which includes its subseries such as Lecture Notes in Artificial Intelligence and Lecture Notes in Bioinformatics, is the most prolific source, with 17 articles published. The "Journal of Neuroscience" follows closely with 13 articles, highlighting its significant role in disseminating research in this domain. Other key sources include "Physica D: Nonlinear Phenomena" with nine articles and both the "Journal of Mathematical Biology" and the "Journal of Physics: Conference Series," each contributing eight articles. Similarly, the "SIAM Journal on Applied Dynamical Systems" has also published eight articles, indicating its importance in the intersection of applied mathematics and neuroscience. The "Annals of the New York Academy of Sciences" and "Biological Cybernetics" each contributed seven articles, showcasing their relevance in the broader scientific discussions within the field. Additionally, the "Journal of Computational Neuroscience" matches this contribution with seven articles, reflecting its focus on computational methods in neuroscience. Lastly, the "ASEE Annual Conference and Exposition, Conference Proceedings" rounds out the list with six articles, underlining the importance of conference proceedings in the dissemination of cutting-edge research. These sources represent key venues for publishing influential research in mathematical neuroscience.

**Table 3:** Most Relevant Sources in Mathematical Neuroscience and Their Number of Published Articles

Sources	Number of articles
Lecture Notes in Computer Science (including Subseries Lecture Notes in Artificial Intelligence and Lecture Notes in Bioinformatics)	17
Journal of Neuroscience	13
Physica D: Nonlinear Phenomena	9
Journal of Mathematical Biology	8
Journal of Physics: Conference Series	8
SIAM Journal on Applied Dynamical Systems	8
Annals of the New York Academy of Sciences	7
Biological Cybernetics	7
Journal of Computational Neuroscience	7
ASEE Annual Conference and Exposition, Conference Proceedings	6

Countries' Scientific Production

Table 4 provides an overview of the scientific production in mathematical neuroscience by country. The United States leads by a substantial margin, contributing 699 articles, reflecting its dominant role in this research area. The United Kingdom follows with 195 articles, and China is close behind with 138 articles, indicating strong research activities in these regions as well. Italy and Germany have made significant contributions, with 79 and 74 articles, respectively. Canada and France also feature prominently, with 68 and 63 articles, respectively, showcasing their active participation in this field. Brazil (49 articles) and Spain (46 articles) represent the leading contributors from Latin America and Southern Europe. Belgium rounds out the top 10 with 41 articles, underscoring its involvement in advancing research in mathematical neuroscience. These figures illustrate the global distribution of research efforts, with a strong concentration in North America, Europe, and China.

**Table 4:** Scientific Production by Country in Mathematical Neuroscience and Number of Published Articles

Region	Number of articles
United States	699
United Kingdom	195
China	138
Italy	79
Germany	74
Canada	68
France	63
Brazil	49
Spain	46
Belgium	41

Trend Topics

**Figure 4:**
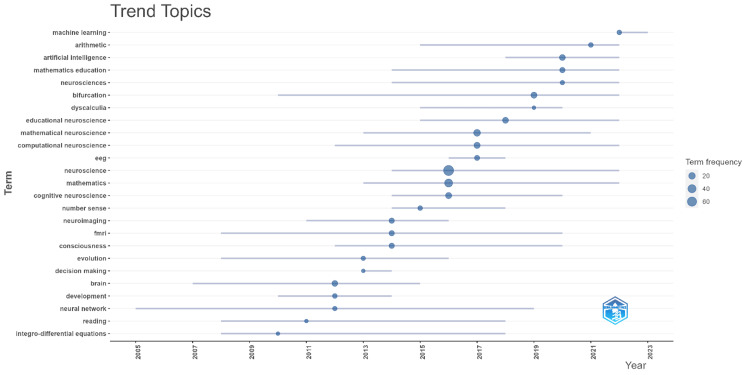
Trends in Research Topics Within Mathematical Neuroscience EEG: electroencephalogram, fMRI: functional magnetic resonance imaging

Figure 4 illustrates the trend topics in mathematical neuroscience, highlighting the evolution and frequency of key terms over time. The terms are represented by circles of varying sizes, with larger circles indicating higher term frequency. From the graph, it is evident that topics such as "machine learning," "arithmetic," and "artificial intelligence" have gained significant prominence in recent years, particularly from around 2017 onward. These topics reflect the increasing integration of advanced computational techniques in mathematical neuroscience. Earlier in the timeline, topics such as "neurosciences," "bifurcation," and "dyscalculia" were more prevalent, suggesting a focus on foundational neuroscience and specific neurological conditions in the early 2000s. As time progresses, there is a clear shift toward more interdisciplinary and technologically advanced topics. Terms such as "educational neuroscience," "mathematical neuroscience," and "computational neuroscience" also show a steady presence, indicating ongoing research interest in applying mathematical and computational methods to understand neural processes and educational outcomes. Emerging topics such as "neural network," "consciousness," and "decision-making" appear to be gaining traction, particularly from 2010 onward, reflecting the broader interest in cognitive neuroscience and its applications. Overall, the trend analysis depicted in Figure 3 provides valuable insights into the evolving landscape of mathematical neuroscience research, with a notable shift toward integrating machine learning and artificial intelligence in recent years.

Thematic Map

**Figure 5:**
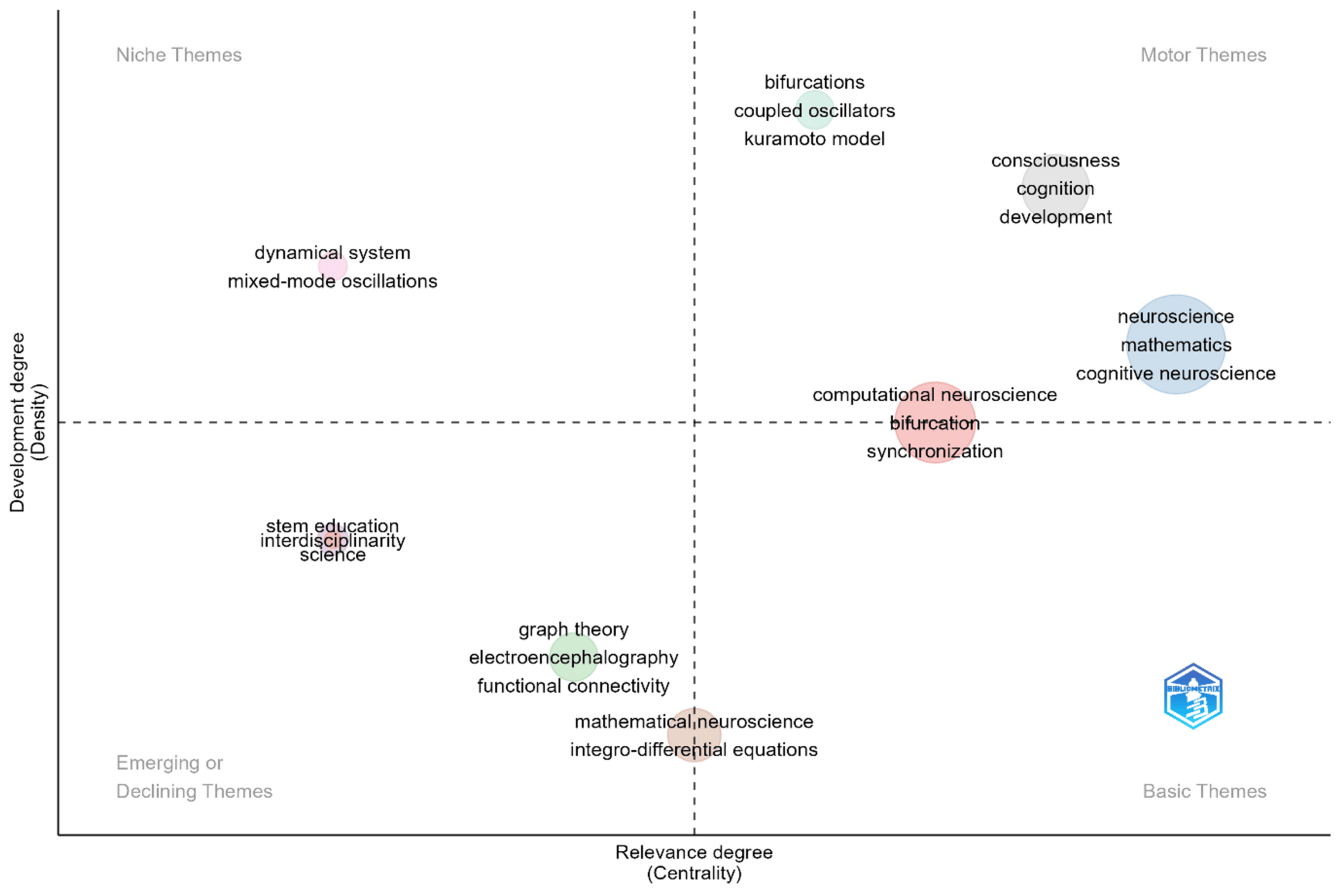
Thematic Map of Research Areas in Mathematical Neuroscience STEM: science, technology, engineering, and mathematics

Figure 5 portrays the thematic map of the research themes in mathematical neuroscience. The themes are charted in this map according to growth or development (density) into four quadrants, where each quadrant is assigned in different relevance, which is computed by centrality measures. In the quadrant of motor themes, we find top themes whose development is high and central, such as "neuroscience," "mathematics," "cognitive neuroscience," "consciousness," "cognition," and "development." These topics identify themselves with the field and are the main areas of research; with this, they signal their fundamental nature and broad application in mathematical neuroscience. The most prominent topics in the upper-left quadrant of this niche map of themes include things such as bifurcations, coupled oscillators, the Kuramoto model, and mixed-mode oscillations, among many others in the general area of dynamical systems. These are all fine, well-developed topics that exist as a thematic core of certain subfields but have not been broadly integrated into the general research landscape. "STEM education," "interdisciplinary science," "graph theory," "electroencephalography," and "functional connectivity" lie in the portion of the quadrants that represents emerging themes or declining themes: those that are at an early stage of development or possibly losing relevance within the discipline. Finally, the basic themes quadrant includes themes such as "computational neuroscience," "synchronization," "bifurcation," and "mathematical neuroscience." These would be regarded as fairly preeminent themes, whereas less dense development is characteristic in comparison with the motor themes quadrant. However, they do have pioneering concepts and are building blocks toward more complex or specialized research. The thematic map proposed in Figure 5 roughly gives a clean view relative to the landscape of research themes in mathematical neuroscience, both the mature, well-focused core areas and the emerging or niche themes to which its front is beginning to broaden.

Three-Field Plot

**Figure 6:**
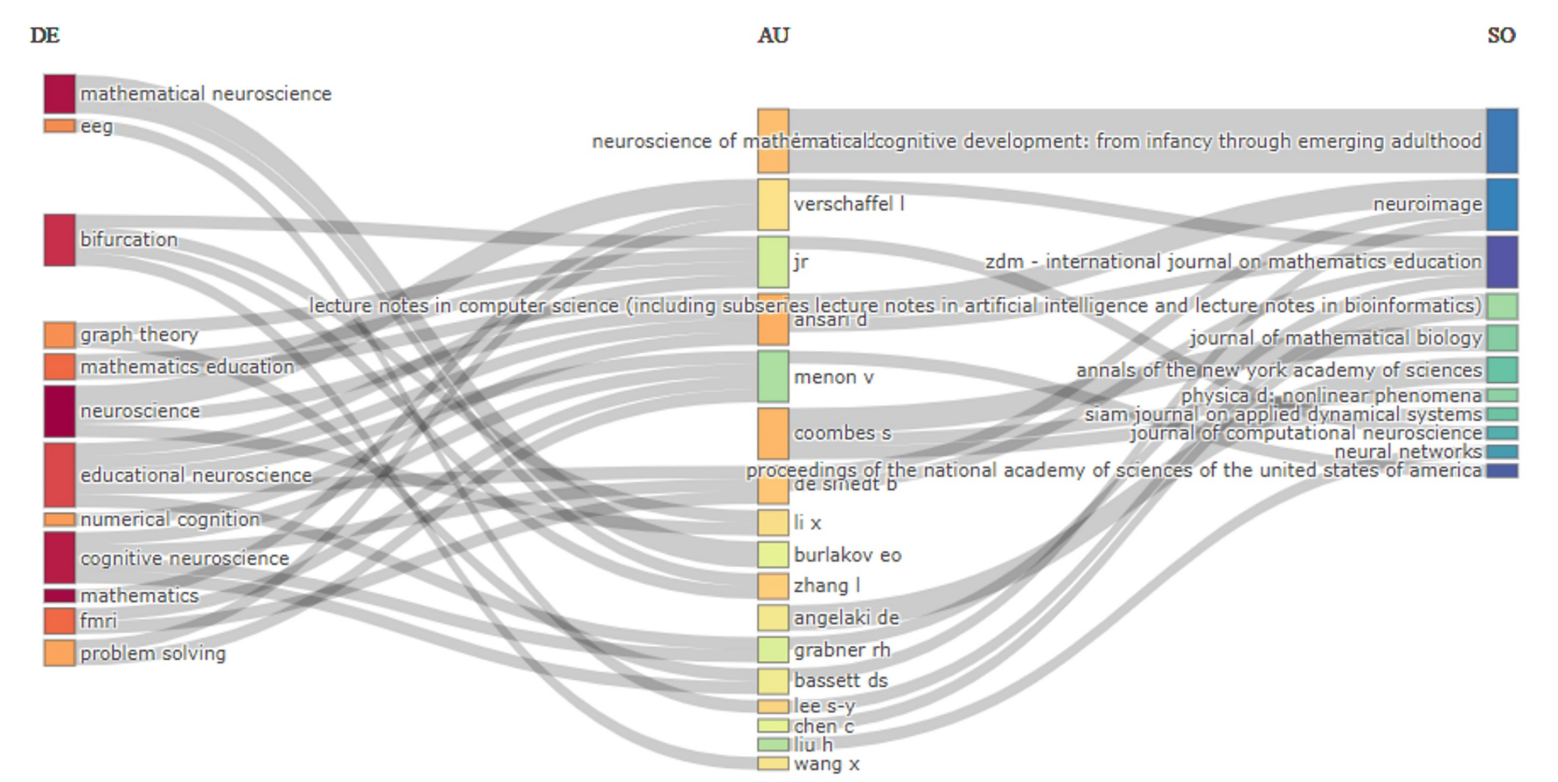
Three-Field Plot Illustrating Connections Between DE, AU, and SO in Mathematical Neuroscience DE: descriptors (keywords), AU: authors, SO: sources (publication sources), EEG: electroencephalogram, fMRI: functional magnetic resonance imaging

The three-field plot in Figure 6 provides an illustration of the links between keywords, authors, and publication sources in the field of mathematical neuroscience. From the plot, one understands the key players, their focus, and where their works are published. On the left, we can see highly visible terms such as "mathematical neuroscience," "eeg," "bifurcation," "neuroscience," and "cognitive neuroscience." These keywords represent the main areas of focus in research covered by the authors listed. In the middle, authors such as Brown RD, Ansari D, Coombes S, and De Smedt B are linked to a number of keywords, thus their wide contribution to the research. Connections between authors and keywords suggest their specific interests. This is understandable, given that some authors are focused on a larger number of topics; however, sources such as Lecture Notes in Computer Science, Journal of Mathematical Biology, and Neuroimage have a high probability of being connected with more specialized authors, as well as keywords; the proportions attest to the kind of role these journals can play as an important outlet for mathematical neuroscience research. The plot shows the cross-discipline nature of the field, where authors are contributing to a wide variety of topics and publish in a number of key journals. Furthermore, it might well be giving an indication that there may be some interlock between particular areas of research represented by keywords and ways in which these are investigated by various researchers mapping out the transmission of knowledge through key publication venues. This visualization can provide guidance about the research landscape and the influential authors, topics, and journals within a domain.

Co-authorship Between Countries

**Figure 7:**
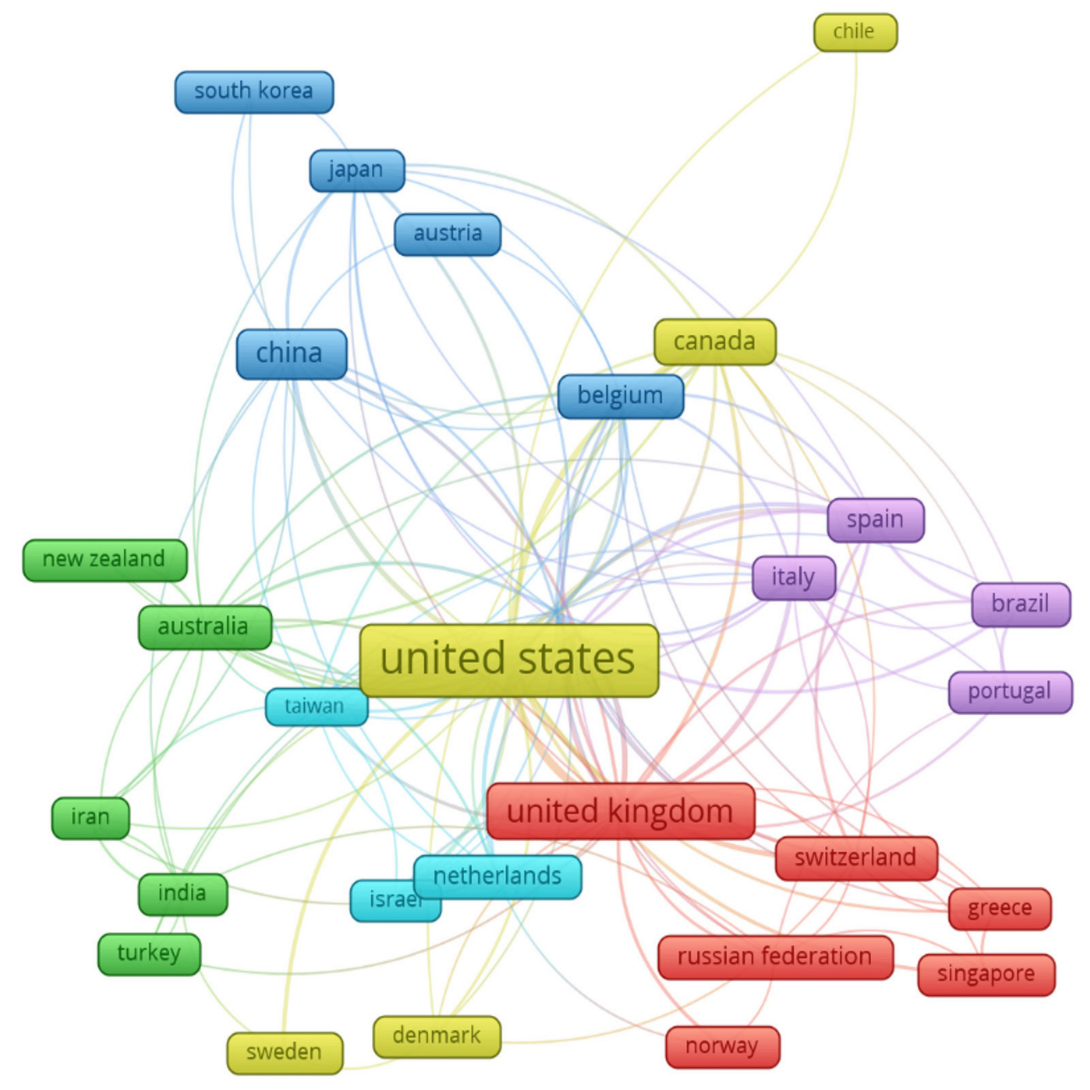
Co-authorship Network Between Countries in Mathematical Neuroscience Node size: The size of each node represents the relative contribution of each country in terms of the number of publications. Larger nodes indicate countries with higher contributions to the field of mathematical neuroscience. Node color: The color of each node groups countries into clusters based on the strength of their collaborative relationships. Countries within the same color cluster tend to collaborate more frequently with each other. Line thickness: The thickness of the connecting lines between nodes represents the strength or extent of collaboration between countries. Thicker lines indicate stronger or more frequent collaborative efforts. Node position: The spatial arrangement of nodes reflects the overall structure of the co-authorship network, where countries positioned closer together have stronger collaborative ties.

Figure 7 illustrates the co-authorship network between countries in the field of mathematical neuroscience. This network map, created with VOSviewer, includes all relationships and collaborations between different countries, in which relative contributions from each country are reflected in the size of its node, while line thickness will represent the strength or extent of collaboration. The United States thus acts as the hub in this network, which further reiterates its dominant contributions to the field. It has strong links of collaboration with some countries, more specifically the United Kingdom, China, Canada, and Australia. This demonstrates a high level of international collaboration necessary for the advancement of research in mathematical neuroscience. The United Kingdom also emerges as a major hub, with maximum links to almost all the European and other countries, including Switzerland, Spain, Italy, and the Russian Federation. This highlights the pivotal role of the United Kingdom in hosting research collaborations within the European continent and across continents. Another major player on the world stage is China, with amazing levels of collaboration with countries such as Japan, South Korea, Australia, and Belgium. The growing prominence of China in this field is reflected in numerous strong ties with a number of Western and Asian countries. Other peripheral roles include those of Canada, Australia, Germany, and Italy, each represented by multiple connections indicating their active participation in international research projects. Smaller nodes, such as Chile, Portugal, Singapore, and Norway, stand for countries with emerging or more focused contributions but still hold pivotal collaborative relations within the international network. In this sense, the co-authorship network map described in Figure 7 emphasizes the necessity and relevance of international collaboration for advances in mathematical neuroscience. These wide-ranging connections underpin the global nature of research efforts, with some countries acting as a bridge between different regions to exchange knowledge and expertise.

Co-occurrence of Keywords

**Figure 8:**
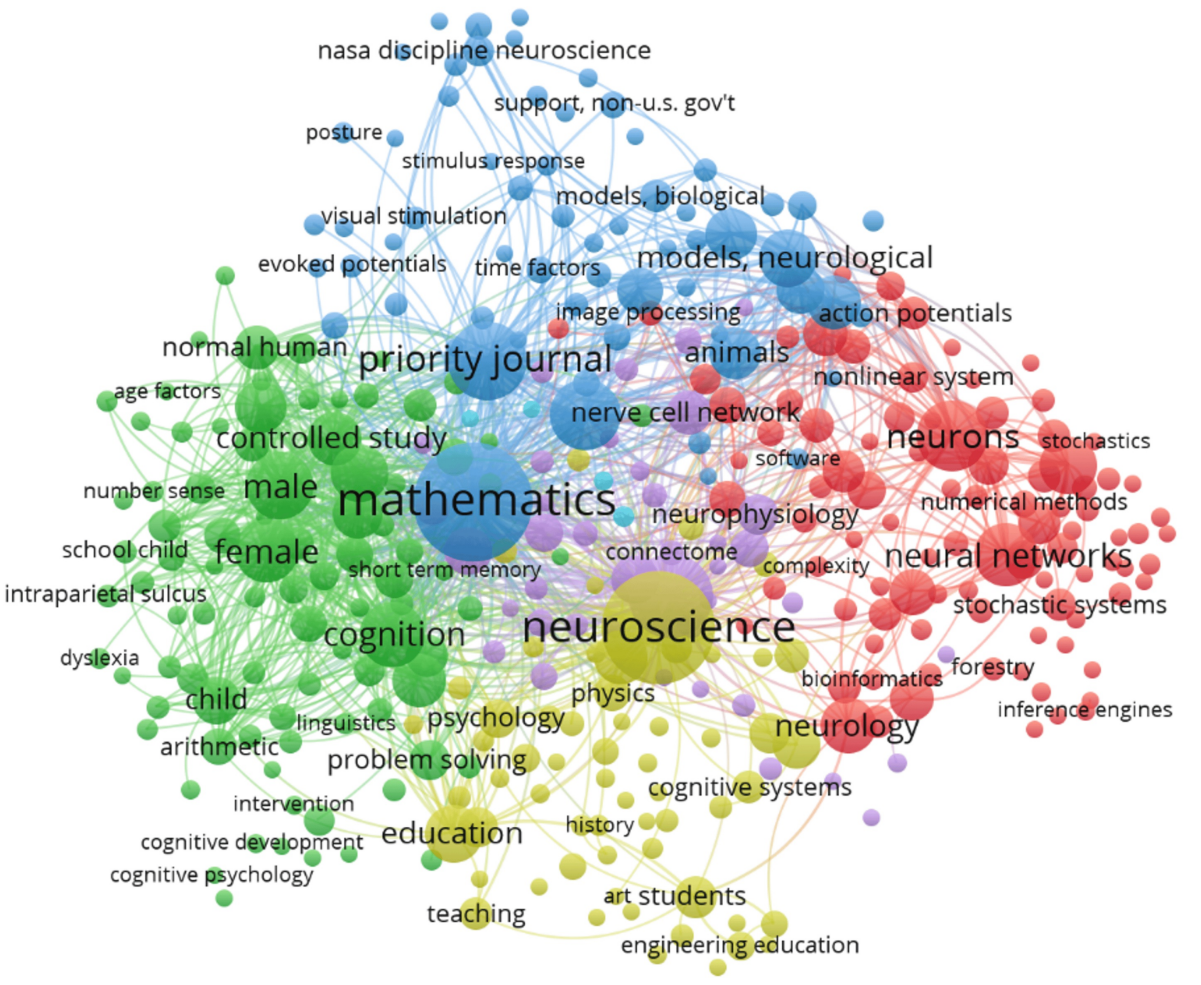
Network Visualization of Keyword Co-occurrence in Mathematical Neuroscience Node size: The size of each node represents the frequency of occurrence of the corresponding keyword in the research documents. Larger nodes indicate keywords that appear more frequently, highlighting central themes in the field. Node color: The color of each node corresponds to different clusters of related keywords. Each color represents a group of keywords that frequently co-occur, suggesting a specific research theme or focus area within mathematical neuroscience. For example, green might represent educational and cognitive aspects, red might indicate computational and neurological aspects, blue might be related to neurological processes, and yellow might be related to educational applications of neuroscience. Line thickness: The thickness of the lines connecting nodes represents the strength of the co-occurrence relationship between keywords. Thicker lines indicate stronger connections, meaning that the connected keywords often appear together in the same research documents. Node position: The spatial arrangement of nodes reflects the overall structure of the co-occurrence network, where keywords positioned closer together have a higher likelihood of appearing together in the literature.

The keyword co-occurrence network for mathematical neuroscience, shown in the VOSviewer map in Figure 8, reveals that quite a number of research themes are strongly interrelated. There are several central themes, such as "mathematics," "neuroscience," and "neural networks," which appear to play fundamental roles in the field because of their high frequency of appearance in the network. These central nodes are directly and strongly linked with the other prominent terms in the map, which are "neurons," "cognition," and "education," hence representing the interdisciplinary nature of research connecting neuroscience with mathematical modeling and its outcome in education. The map comprises a number of clusters based on the focuses of research. The green cluster focuses on educational and cognitive aspects, with such terms as "cognition," "controlled study," and "arithmetic." In all, 11 of these articles show a strong connection of mathematical concepts with the educational course of cognitive development. The red cluster tends to relate to computational and neurological aspects of the topic; here are keywords such as "neurons," "neural networks," and "stochastics," which pioneers a mathematical framework of neural mechanisms. The blue cluster is about demonstrations of neurological processes. Key terms include "models," "neurological," and "biological models," which describe activities related to the simulation and understanding of biological systems. In the yellow, neuroscience is theater for pedagogy; one can find "education" and "cognitive systems," indicating the application of neuroscience in educational practice. As a whole, the map underlines that this mathematical neuroscience is very interdisciplinary. Its foundational concepts interlink to provide contrast with applied research in education, computational modeling, and cognitive psychology. On the other hand, dense interconnections between different clusters suggest a very collaborative and integrative research landscape.

Bibliographic Coupling Between Countries

**Figure 9:**
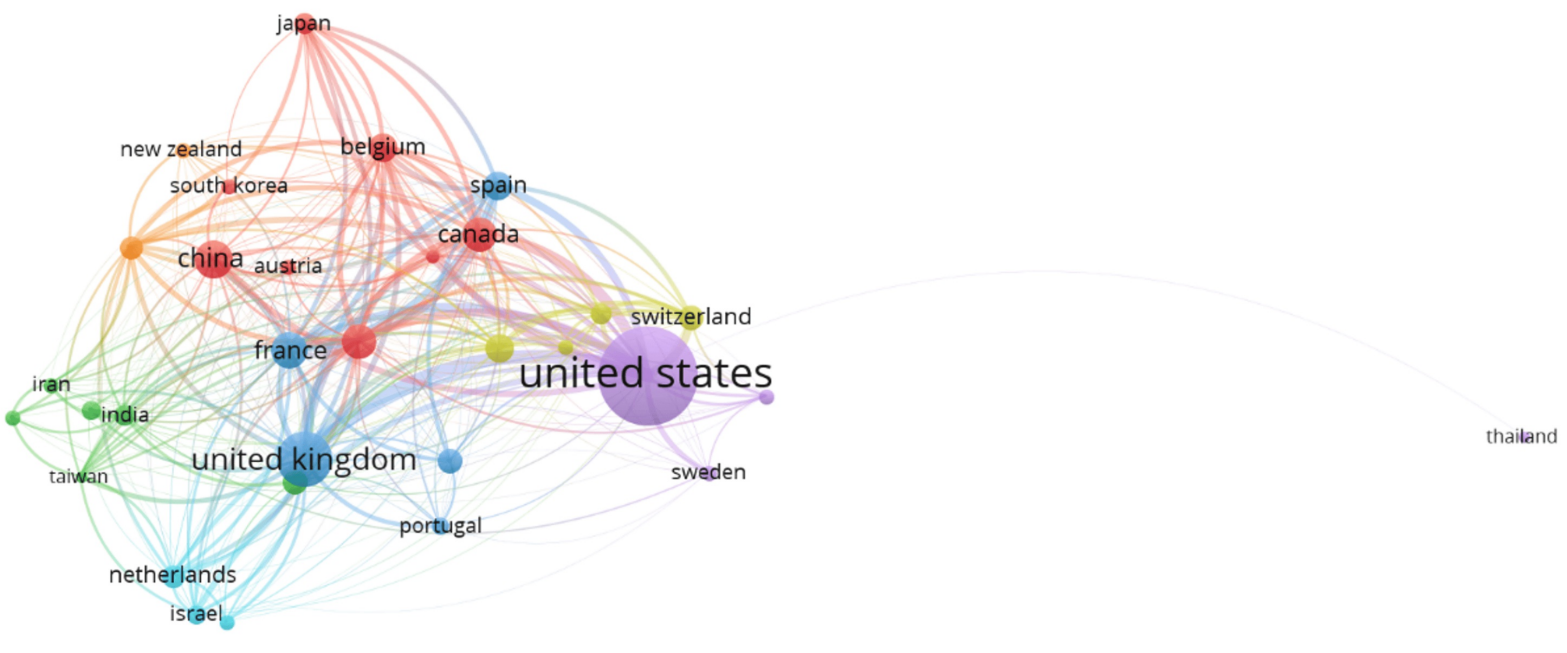
Bibliographic Coupling Network Between Countries in Mathematical Neuroscience Node size: The size of each node represents the volume of research output from each country in the field of mathematical neuroscience. Larger nodes indicate countries with higher research output and a stronger presence in the global research landscape. Node color: The color of each node corresponds to different clusters of countries that share strong bibliographic coupling. Countries within the same color cluster tend to cite similar documents, reflecting common research interests or thematic alignment. Line thickness: The thickness of the lines connecting nodes represents the strength of the bibliographic coupling between countries. Thicker lines indicate that the connected countries frequently cite the same documents, showing a high level of research alignment or collaboration through shared references. Node position: The spatial arrangement of nodes indicates the relative proximity in bibliographic coupling strength. Countries positioned closer together share a greater number of cited references, suggesting stronger intellectual connections.

Figure 9 shows the bibliographic coupling among countries working in mathematical neuroscience. It is evident that many countries are strongly connected by their references. Bibliographic coupling occurs whenever two or more countries have cited the same documents, thus showing some research alignment or even collaboration. The United States is the largest node, which shows its centrality in the global research landscape of mathematical neuroscience. It has a strong bibliographic coupling with many countries, such as the United Kingdom, China, Japan, Canada, and France. That means the United States not only produces a large amount of research but also shares a large number of references with these countries, indicating close research ties. The United Kingdom and China also represent prominent nodes, thus indicating their large contribution to the area and strong bibliographic links with other leading countries. Particularly, the United Kingdom has huge links with European countries such as France, Netherlands, Belgium, and Spain, and with Japan and Australia. The case of Japan, in this respect, is represented as a hub of this network, an intense bibliographic coupling with countries in Europe, North America, and Asia, denoting its role as a bridge across the global research community. Switzerland and Sweden also stand as strong linkages, mostly to other European countries and the United States, toward robust participation in international research efforts. In contrast, Thailand acts as a more isolated node with fewer connections, which could be interpreted to mean that it is more independent or even an up-and-coming presence within the field, with less bibliographic coupling as opposed to the more established research nations. Figure 9 thus gives the bigger picture regarding the connectedness of the world research community working in mathematical neuroscience, where dense networks made up of shared references by some key countries are indicative of close research collaboration and integration. The map underlines the requirement for international cooperation to advance the field, wherein a few leading countries will act as interlinking centers among various regional research efforts.

Discussion

These findings provide a general overview of the research landscape for mathematical neuroscience from 1973 to 2024, covering major trends in the field, influential authors, important publications, and collaborative networks. Several interesting observations from this analysis add significantly to our knowledge about how mathematical neuroscience has evolved and where it may be headed in the future.

Probably one of the most striking results is that the annual growth rate for publications equals 6.51%, which indicates steady yearly growth of research activity over the last five decades. This surge is especially well noticeable during the early 2000s, when technological advancement and growing acknowledgment of the need for interdisciplinary approaches likely fueled this surge. The fact that there are peaks of publication activity in 2014, 2016, and 2020 may indicate that some events or developments within the periods have had important influences on the research field.

The analysis of the most relevant authors, such as Ansari D and Coombes S, underlines the contribution of key people in shaping the direction of mathematical neuroscience. Such individuals have contributed a large number of publications and, importantly, a large number of citations, which reflects their influence and impact on the academic community. Finally, author production over time puts the evolution of authors' contributions in a different perspective: some researchers kept a steady output over many years, while many others have more concentrated periods of activity.

Most of the publications on mathematical neuroscience are covered by a few sources, journals, and conference proceedings. The "Lecture Notes in Computer Science" series, "Journal of Neuroscience," and "Physica D: Nonlinear Phenomena" capture a significant share of publications in the area, indicating its interdisciplinary nature, touching upon neuroscience, mathematics, and computational sciences. This interdisciplinary approach is further evidenced by the thematic map, which categorizes issues in the field into primary research themes, niche topics, and emerging or declining areas.

The country-level analysis on scientific production and co-authorship networks underlines the global nature of mathematical neuroscience research. Especially, the United States had become a driver of this research field by the amplitude of its contribution and central role in international collaboration. The close links between the United States, the United Kingdom, China, and other countries highlight the actual need for cross-border collaboration in order to advance knowledge and be innovative. The analysis also, however, reveals that there has been a shift toward such countries as China taking a growing share, reflecting the international growth in research in mathematical neuroscience.

Through co-occurrence and bibliographic coupling analysis, the intellectual structure and collaborative networks in the field can be revealed. The co-occurrence of keywords seems to depict that mathematical neuroscience is highly interdisciplinary, bringing together basic areas of "neural networks" and "cognition" with application research areas on education and computational modeling. On the other hand, the bibliographic coupling map reveals highly connected research across different countries: leading countries form dense networks of shared references, indicative of high research alignment and cooperation.

This research provides an in-depth review of trends, major contributors, and collaborative networks in the field of mathematical neuroscience. Results accentuate incredible growth in this field; the interdisciplinary nature of methods used in problems and the high relevance of international collaboration in driving the frontier are brought into view. With this view, a fairly meticulous follow-up of these trends, mostly in the aspect of integrating emergent technologies such as machine learning and the application of artificial intelligence, is highly expected to prepare the future of mathematical neuroscience.

Research Gaps

While this analysis provides a comprehensive overview of the research landscape in mathematical neuroscience, several gaps remain that require further investigation. One significant gap is the limited exploration of regional research disparities. Although this study identifies global research trends, it lacks an in-depth analysis of the unequal contributions across regions, particularly the growing influence of countries such as China, which is becoming an increasingly significant player in the field. A deeper examination of how countries with different levels of research output engage in international collaboration, as well as their impact on the field, would provide valuable insights into global scientific dynamics.

Additionally, the study has focused primarily on quantitative assessments of publication trends, influential authors, and key themes, without an in-depth qualitative analysis that considers methodological challenges or theoretical advancements driving the field. Investigating these aspects could enhance the understanding of underlying trends and help highlight emerging research frontiers. The study also touches on the underrepresentation of interdisciplinary areas, particularly in bridging mathematical neuroscience with fields such as artificial intelligence (AI), cognitive science, or neuroeducation. Exploring these intersections could shed light on new, underexplored research areas. Finally, while the study provides a broad view of research trends, it does not fully examine the potential effects of recent technological advancements or major global events, such as the COVID-19 pandemic, on the evolution of research priorities and international collaboration in this field.

Practical Implications

The findings of the research have crucial practical implications for researchers, educators, and policymakers working in mathematical neuroscience. It is hoped that the identification of key trends of research, influential authors, and leading publication venues turns out to be useful pointers to guide further research efforts and collaborations. The steady growth of publications and increasing prominence of interdisciplinary topics, such as machine learning and computational neuroscience, signal a clear demand for the incorporation of sophisticated computational tools and methods into neuroscience research and training. Furthermore, the global spread of research underscores the need for international collaboration on transborder research programs and policies to further this field. Understanding the evolving landscape in mathematical neuroscience will hence be important not only in setting an agenda for curriculum development and research funding priorities but also in knowledge dissemination toward broader application for use in understanding complex neural processes using mathematical models.

## Conclusions

A cross-discipline of mathematics, computational techniques, and neuroscience, mathematical neuroscience developed drastically over the past few decades. This paper tries to identify some trends of key research conducted in the current field: one is that advanced computational methods have been increasingly applied to understand complicated neural processes, and another important point is that global collaboration plays a key role while considering progressive research. The most influential contributors and countries related to this field are also identified. It is an evolving research landscape, and it is clear that foundational areas such as cognitive neuroscience and computational modeling remain at the heart, with new topics and methods coming up. International partnerships are critical to drive innovation and progress, as characterized by the depth of co-authorship and shared research networks. The further the field develops, the more research will most certainly take new directions in the future and become increasingly integrated with advanced technologies in the quest to understand brain functions and cognitive processes. The present study lays down invaluable input that may direct active research and support acquired improvements in the field.
